# Clinical and epidemiological baseline characterization of dementia cases in a specialized center in Recife

**DOI:** 10.1002/alz.093184

**Published:** 2025-01-09

**Authors:** Mariana Albuquerque de Luna, Camila Oliveira Negri, Bruna Cristina de Oliveira Lima, João Augusto de Macedo Cavalcanti de Albuquerque, Bárbara Nascimento Moutinho, Ilo de Vasconcelos Ximenes Júnior, Mariana Leandro Silva, Tatiana Caldas Neves da Silva, Barbara Matos Almeida Queiroz, Breno José Alencar Pires Barbosa

**Affiliations:** ^1^ Universidade Federal de Pernambuco, Recife Brazil

## Abstract

**Introduction:**

Dementia numbers are increasing rapidly with a substantial contribution from low and middle‐income countries. There’s a limited number of reports in Brazil, mostly modest initiatives from south‐eastern, more developed regions. With a population of approximately 1.6 million individuals, Recife is among the largest cities of Northeast Brazil, a region of steep social disparities and high prevalence of low‐literacy. The aim of this study was to describe the sample of patients followed at a dementia outpatient clinic in a tertiary center in Recife

**Methods:**

this is a single‐center, descriptive and health records‐based analysis of patients evaluated in the dementia outpatient clinic of the Neurology and Neurosurgery Unit at the Federal University of Pernambuco between 2018 – 2023.

**Results:**

168 medical records were analyzed, of which 137 met the inclusion criteria. 44.59% were male, with a mean age of 66 years. 29.3% of the sample had 8 to 11 years of formal education. On the other hand, 21.7% were illiterate. The mean baseline score on the Mini‐Mental score was 17. Alzheimer’s disease was the most frequent diagnosis (25%), followed by frontotemporal dementia (9.46%), Lewy body disease (4.05%) and Pure Vascular dementia (4.05%). The woman’s main caregiver was her offspring (79%). The man´s main caregiver was his partner (84%). 44% of the sample had hypertension and diabetes (25%). 20% were alcoholic and 19% were smokers. The average follow‐up time for severe dementia was 30.4 months. 66.4% of our sample underwent magnetic resonance imaging and 16% had access to molecular imaging methods.

**Conclusion:**

patients referred for evaluation were mostly in moderate to advanced stages of dementia, with a significant prevalence of mixed‐type and non‐Alzheimer’s pathologies. This data could suggest a referral bias and poor access to specialized clinics. Despite a modest sample size and heterogeneous data, the present study may contribute towards a better characterization of dementia numbers in the Northeast of Brazil.

